# Projecting species’ vulnerability to climate change: Which uncertainty sources matter most and extrapolate best?

**DOI:** 10.1002/ece3.3403

**Published:** 2017-09-20

**Authors:** Valerie Steen, Helen R. Sofaer, Susan K. Skagen, Andrea J. Ray, Barry R. Noon

**Affiliations:** ^1^ Department of Fish, Wildlife and Conservation Biology Colorado State University Fort Collins CO USA; ^2^ U.S. Geological Survey Fort Collins Science Center Fort Collins CO USA; ^3^ NOAA/Earth System Research Laboratory Boulder CO USA

**Keywords:** bioclimatic species distribution models, climate change vulnerability, climate covariates, collinearity, general circulation models, Prairie Pothole Region, projection uncertainty, thresholds, wetland‐dependent birds

## Abstract

Species distribution models (SDMs) are commonly used to assess potential climate change impacts on biodiversity, but several critical methodological decisions are often made arbitrarily. We compare variability arising from these decisions to the uncertainty in future climate change itself. We also test whether certain choices offer improved skill for extrapolating to a changed climate and whether internal cross‐validation skill indicates extrapolative skill. We compared projected vulnerability for 29 wetland‐dependent bird species breeding in the climatically dynamic Prairie Pothole Region, USA. For each species we built 1,080 SDMs to represent a unique combination of: future climate, class of climate covariates, collinearity level, and thresholding procedure. We examined the variation in projected vulnerability attributed to each uncertainty source. To assess extrapolation skill under a changed climate, we compared model predictions with observations from historic drought years. Uncertainty in projected vulnerability was substantial, and the largest source was that of future climate change. Large uncertainty was also attributed to climate covariate class with hydrological covariates projecting half the range loss of bioclimatic covariates or other summaries of temperature and precipitation. We found that choices based on performance in cross‐validation improved skill in extrapolation. Qualitative rankings were also highly uncertain. Given uncertainty in projected vulnerability and resulting uncertainty in rankings used for conservation prioritization, a number of considerations appear critical for using bioclimatic SDMs to inform climate change mitigation strategies. Our results emphasize explicitly selecting climate summaries that most closely represent processes likely to underlie ecological response to climate change. For example, hydrological covariates projected substantially reduced vulnerability, highlighting the importance of considering whether water availability may be a more proximal driver than precipitation. However, because cross‐validation results were correlated with extrapolation results, the use of cross‐validation performance metrics to guide modeling choices where knowledge is limited was supported.

## 1 INTRODUCTION

Bioclimatic species distribution models (SDMs) are useful tools for assessing the potential impacts of climate change on biological diversity (Barbet‐Massin, Walther, Thuiller, Rahbek, & Jiguet, 2009; Lawler et al., 2009; Thuiller, Lavorel, Araujo, Sykes, & Prentice, 2005). These models relate species location data to climate covariates to derive probabilities of occurrence over a range of climatic conditions. When applied to future climate scenarios, the degree of any overall contraction in projected species’ distributions can be used to indicate a species’ vulnerability to future climate‐mediated population declines (Dawson, Jackson, House, Prentice, & Mace, 2011; Glick, Stein, & Edelson, 2011). Various methodological decisions are an inherent part of model development and some have been shown to produce large uncertainty, often exceeding the uncertainty in future climate change itself (Buisson, Thuiller, Casajus, Lek, & Grenouillet, 2010; Garcia, Burgess, Cabeza, Rahbek, & Araujo, 2012; Synes & Osborne, 2011; Thuiller, 2004). Understanding the relative importance of different uncertainty sources—and how to improve model performance—can help guide allocation of effort in sensitivity analyzes and improve projections of species’ vulnerability to climate change.

Attention to methodological decisions has often focused on comparisons of statistical models (e.g., Heikkinen et al., 2006), a decision now often supplanted by the use of statistical model ensembles (Araujo, Whittaker, Ladle, & Erhard, 2005). Some attention has been directed toward the selection of climate covariates (Gaston, 2003; Pliscoff, Luebert, Hilger, & Guisan, 2014; Synes & Osborne, 2011), but many studies are nevertheless based on a small set of easily accessible and generic climate variables (e.g., bioclim variables from the Worldclim dataset; Hijmans, Cameron, Parra, Jones, & Jarvis, 2005). Recent work has highlighted decisions regarding temporal scale and type of climate variable summaries (van de Pol et al., 2016), but there remains a broad need for comparison among classes of climate variables, including hydrological variables that integrate the effects of temperature and precipitation to assess the impacts of climate change on water availability (Austin, 2007). Furthermore, when selecting variables, researchers must decide on the appropriate tolerance for collinearity. While a common practice is to limit collinearity and thus limit the potential for overfitting (Beaumont, Hughes, & Poulsen, 2005; Dormann et al., 2013), it has also been suggested that changes in future covariance structure and the potential for selecting a noncausal variable may favor retaining collinear covariates (Braunisch et al., 2013). Assessing whether performance in cross‐validation is indicative of performance in extrapolation (i.e., in new times, places, or conditions) is a key step for guiding these choices, and has not been widely explored for these nor for additional decisions such as the method used to threshold continuous model predictions to create binary maps of species distributions. Comparing the magnitude of uncertainty deriving from methodological choices to the magnitude of uncertainty deriving from variability in projections of future climate can provide researchers with a guide for how to allocate modeling effort—that is whether greater consideration should be given to capturing the uncertainty in future climate or the uncertainty due to methodological choices.

We evaluated the uncertainty in projections of climate‐driven distributional changes arising from different methodological decisions, and evaluated whether cross‐validation led to selection of the choices that maximized performance during pronounced historical drought periods, a proxy for future climate change. We focused on wetland‐dependent birds breeding in the Prairie Potholes of North America, a climatically dynamic landscape where climate change may reduce the abundance and productivity of wetlands that provide critical habitat for wetland‐dependent species (Johnson et al., 2010; Sorenson, Goldberg, Root, & Anderson, 1998; Steen, Skagen, & Noon, 2014). The distribution of migratory birds is reflective of the current year's wetland conditions (Fletcher & Koford, 2004; Johnson & Grier, 1988; Niemuth & Solberg, 2003; Smith, 1970), allowing for inference into climatic effects on avian occurrence patterns. We used SDMs trained on normal and wet climatic conditions and predicted occurrence under drought to evaluate model performance when projecting to a climatically nonstationary period; future climate projections point to increasing summer drought in this system (Ballard et al., 2014). We address the following questions:

1. What is the amount of uncertainty in projections of range change attributable to a) GCM; b) climate covariate class; c) degree of collinearity; and d) thresholding procedure?
2. How is the amount of projected range change affected by choices of a) GCM; b) climate covariate class; c) degree of collinearity; and d) thresholding procedure?
3. Which choices provide the best ability to extrapolate to a drought period? Are the same choices recommended based on cross‐validation?

## 2 METHODS

### 2.1 Study system and data sources

The Prairie Pothole Region (PPR) is an extensive freshwater wetland ecoregion in North America that hosts numerous breeding wetland‐dependent bird species, including waterfowl species that have made the region a focus for conservation and management efforts (Doherty, Ryba, Stemler, Niemuth, & Meeks, 2013; Ringelman et al., 2005; Steen, Skagen, & Melcher, 2016). This region is characterized by high climatic variability across years, and periods of drought or excessive precipitation may extend over multiyear periods. We obtained species occurrence (presence and absence) data from the North American Breeding Bird Survey (BBS; Sauer et al., 2011) for our focal species for the U.S. portion of the PPR in North Dakota, South Dakota, and Minnesota (See Fig. S1 and Table S1 in Supporting Information). We included land cover covariates to associate with BBS routes that described wetland and upland coverages. We estimated models based on observed climatic conditions (Maurer, Brekke, Pruitt, & Duffy, 2007). To assess climate change impacts we compared future distributions under forecasted climate to distributions under simulated past climate (“hindcast”), an approach that avoids conflating climate model biases with the impacts of climate change (Sofaer et al., 2017). Climate forecasts and hindcasts were obtained from 10 randomly selected CMIP5 GCMs under Representative Concentration Pathway 8.5 (See Fig. S2 in Supporting Information). Hydrological data were output from a macroscale hydrologic model (Liang, Lettenmaier, Wood, & Burges, 1994). Additional information on the Prairie Pothole ecosystem, the study area, and BBS, land cover and climate data sources are in Appendix S1 in Supporting Information.

### 2.2 Climate covariate hypotheses

We devised three classes of climate covariate which can be considered working hypotheses for how climate explains organismal distribution: temporal, bioclimatic, and hydrological. Temporal covariates are based on simple summaries of monthly precipitation and temperature data. We propose the temporal hypothesis as a general hypothesis that precipitation and temperature drive organismal distribution via multiple temporal scales and the variability therein and can include monthly, seasonal, annual, or longer term summaries and variability. The bioclimatic hypothesis proposes that organismal distribution is primarily described by monthly or quarterly extremes and seasonality of temperature and precipitation. The original set of bioclimatic covariates was developed to describe process‐based climatic relationships with plant growth (Booth, Nix, Busby, & Hutchinson, 2014), but bioclimatic covariates now are widely used to describe the distribution of animal species as well (e.g., Elith, Kearney, & Phillips, 2010; Green et al., 2008; Gregory et al., 2009; Jimenez‐Valverde et al., 2011; Lawler, White, Neilson, & Blaustein, 2006). We propose the hydrological hypothesis as a hypothesis that organismal distribution is primarily limited by water availability. Because precipitation is not a reliable representation of water availability and many ecological processes are water limited, hydrological variables may more directly relate to ecological response (McEvoy et al., 2016). Drought indices or soil moisture, for example, have been explored by some researchers (Barbet‐Massin & Jetz, 2014; Crimmins, Dobrowski, Greenberg, Abatzoglou, & Mynsberge, 2011; Konar, Todd, Muneepeerakul, Rinaldo, & Rodriguez‐Iturbe, 2013; Schlaepfer, Lauenroth, & Bradford, 2012), but they are not widely used.

We developed candidate covariate sets for each of the three climate hypotheses based on previous bird and PPR studies (See Appendix S1 and Table S2 in Supporting Information). Candidate covariates for each of the three hypotheses were reduced in number based on their degree of collinearity as assessed by variance inflation factors (VIFs; Table S2). The first VIF cutoff was set at the commonly recommended value of ten, and the second was set at two, using a more stringent recommendation for ecological studies (Kutner, Nachtsheim, & Neter, 2004; Zuur, Ieno, & Elphick, 2010).

### 2.3 Models and vulnerability assessment

We fit occurrence data for 29 wetland‐dependent bird species using the ensemble modeling platform, BIOMOD, implemented in the R package Biomod2 (Version 3.3‐7; Thuiller, Lafourcade, Engler, & Araujo, 2009). We developed consensus predictions based on a weighted probability of occurrence across seven modeling algorithms. To threshold continuous probabilities into binary occurrence values, we used a comprehensive 12 thresholding procedures. To assess climate change vulnerability, we calculated the commonly used Range Change Index (RCI; Thuiller et al., 2005; Buisson et al., 2010; Synes & Osborne, 2011; Fordham, Akcakaya, Araujo, Keith, & Brook, 2013). Additional information on the SDMs, the thresholding procedures, and RCI is in Appendix S1 in Supporting Information.

#### 2.3.1 Attributing uncertainty in range change projections

**Figure 1:**
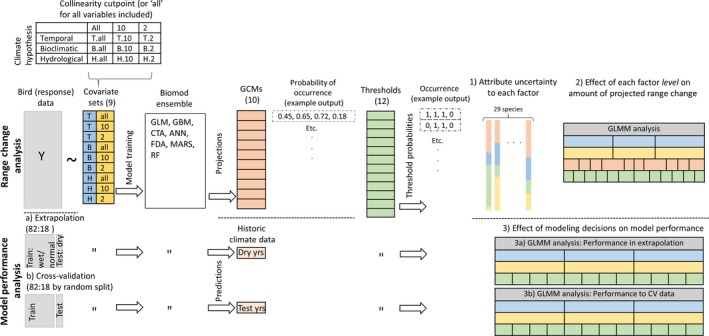
Representation of workflow starting with creating nine species distribution models (SDMs) by training with each of nine covariate sets using the Biomod ensemble and then conducting a *range change analysis* by projecting to future climate (general circulation model data; GCM) or conducting a *model performance analysis* by predicting to testing data. The range change analysis involved projections of each SDM to 10 GCMs to obtain future probabilities of occurrence, then thresholding each projection 12 different ways to obtain occurrence (0/1) values, then calculating range change (range change index; RCI) based on the difference between future versus hindcast occurrence (not shown). The model performance analysis involved predictions of each SDM to subsets of historical climate data based on (a) extrapolation data split or (b) cross‐validation (CV) data splits to obtain historic probabilities of occurrence, then thresholding each prediction 12 different ways to obtain occurrence values, and then assessing model performance based on predicted versus actual occurrence. We used the two types of analyses to address three questions: (1) how much uncertainty is attributable to each uncertainty source (GCM, hypothesis, collinearity, and threshold)? (2) what is the effect of each decision on amount of projected range change?; and (3) what is the effect of modeling decisions on model performance in extrapolation and compared to CV? For objectives 1 and 2, with 1,080 (3 × 3 × 10 × 12) projections per species, we (1) modeled deviance in RCI explained by each uncertainty source, and, (2) modeled the contributions to RCI for the 28 (3 + 3 + 10 + 12) decisions. For the 108 (3 × 3 × 12) historical predictions per species, we modeled the relationship across the 18 (3 + 3 + 12) decisions with model performance in extrapolation (3a) and cross‐validation (3b)

Using a factorial design to evaluate key sources of uncertainty in SDM development, we evaluated all possible combinations of uncertainty from 10 GCMs, three covariate hypotheses, three cut‐offs for collinearity, and 12 thresholding procedures (Figure 1). The result was 1,080 sets of RCI projections per species. We applied a GLM with normal error distribution to log‐transformed RCI output for each species to evaluate the relative contribution to estimated range change arising from GCM selection, covariate hypothesis, degree of collinearity, and thresholding procedure. We alternately withheld each uncertainty source to assess the proportional reduction in model deviance attributable to its inclusion as a model covariate (Buisson et al., 2010). For example, the proportion of deviance explained by GCM, for a given species, was calculated as the difference between the deviance remaining in the model without GCM and the deviance remaining in the model with all uncertainty sources. This difference was then divided by the null (intercept only) model deviance. We then summarized the distribution of deviance reduction values across species for each uncertainty source.

#### 2.3.2 Effects of modeling decisions on amount of projected range change

We assessed the effects of decisions regarding the covariate hypothesis, degree of collinearity, and thresholding procedure on estimates of range change using generalized linear mixed models (GLMMs; Figure 1). In these models, we treated species as a random effect and assumed RCI to be a log normally distributed response variable. GLMMs were created using the R package lme4 (Bates, Machler, Bolker, & Walker, 2015; R Development Core Team, 2012). We set reference levels to those that predicted the smallest RCI. To qualify the degree of change to RCI estimates produced by the alternate decisions compared to choosing the reference level, we describe “none,” “low,” “moderate,” “high,” or “very high” reflecting coefficient estimates of 0, <0 to −0.2, <−0.2 to −0.4, <−0.4 to −0.6, and <−0.6 to −0.8, respectively.

#### 2.3.3 Effects of modeling decisions on model performance when extrapolating

To create independent test data to serve as a proxy for future climate change, we partitioned drought years from the years representing wet and normal conditions (Figure 1). We defined seven drought years: 1988–1992 and 2004–2005. The years 1988–1992 cover a drought considered second in severity only to the dust bowl drought of the 1930's and resulted in a greatly reduced number of wetlands including the loss of some lakes (Niemuth, Wangler, & Reynolds, 2010; Winter & Rosenberry, 1998). During the 2004–2005 drought years, lakes remained largely unaffected but the number of temporary and seasonal wetlands were reduced to below half their maximum number, and sizes of semipermanent wetlands were reduced by ~50% (Niemuth et al., 2010). Extrapolation model performance was assessed using models trained with data from the 82% of the data that represented wet/normal years and projected to the 18% of the data that represented drought years. Cross‐validation model performance was assessed using 10 randomized splits of the wet/normal years’ data using the same proportions as for extrapolation (82:18). We assessed model performance using Cohen's kappa statistic (kappa), True Skill Statistic (TSS), Area under the receiver operating characteristic curve (AUC), and prevalence match (additional information in Appendix S1 in Supporting Information). For modeling purposes, all metrics were logit transformed, and a normal error distribution was assumed (Warton & Hui, 2011).

We modeled the effects of the climate covariate hypothesis, degree of collinearity, and thresholding procedure on model performance for each performance metric (Figure 1). We used GLMMs with species as a random effect (using lme4). For the climate covariate hypothesis and thresholding procedure, we selected reference levels for the GLMMs based on those that were intermediate in effect, thus allowing other levels to have a positive or negative effect on model performance relative to the reference. Using model coefficients, we qualified the relative impact to model performance in extrapolation owing to alternate decisions relative to the intermediate reference level. Therefore, we describe “positive,” “intermediate,” or “negative” performance impacts corresponding to coefficient estimates that were positive, nonsignificant, or negative, respectively.

To evaluate the ability of choices based on higher performance in cross‐validation tests to improve performance for extrapolation as well, we assessed the correlation between cross‐validation and extrapolation results for each performance metric. For each metric, we used GLMMs to predict the extrapolation performance value using a fixed effect of cross‐validation performance value and a random effect of species. We then assessed the correlation between the prediction and the actual extrapolation performance value using Spearman's rank‐based correlation (ρ).

We assessed influential species as diagnosed by the relative variance change measure in the R package HLMdiag by alternately removing each species and observing whether interpretation of model results changed (Dillane, 2006). However, because their removal did not impact the interpretation of the results, we retained them (for the list of influential species, see Appendix S1 in Supporting Information).

Finally, we plotted the distribution of vulnerability ranks across species; this provides a measure of the degree to which modeling decisions affect implications for conservation prioritization (Wright, Hijmans, Schwartz, & Shaffer, 2015).

## 3 RESULTS

### 3.1 Attributing uncertainty in range change projections

**Figure 2:**
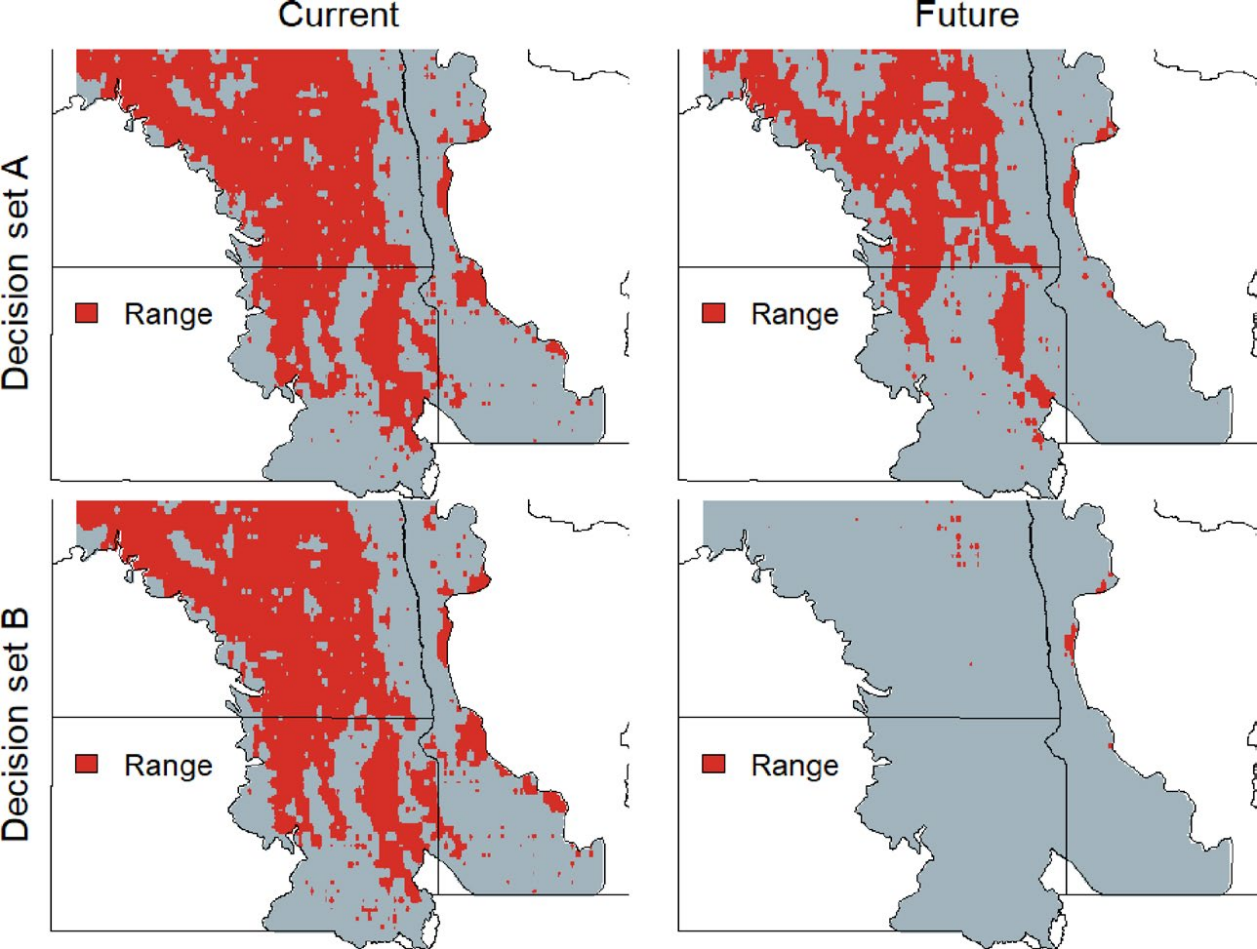
Example comparison of differences in range change for one species (Mallard) under two sets of decisions. Red pixels show locations where species is predicted to occur; the total red area is the range of the species; and thus, range change is the change in red area (future minus current). Decision set A is modeled using: hydrological covariates, variance inflation factor of two to reduce collinearity, kappa threshold, and General Circulation Model “mri.” Decision set B is modeled using: bioclimatic covariates, all covariates, predprevobs threshold, and General Circulation Model “acc.” “Current” is based on General Circulation Model hindcasts rather than observed climate data

**Figure 3:**
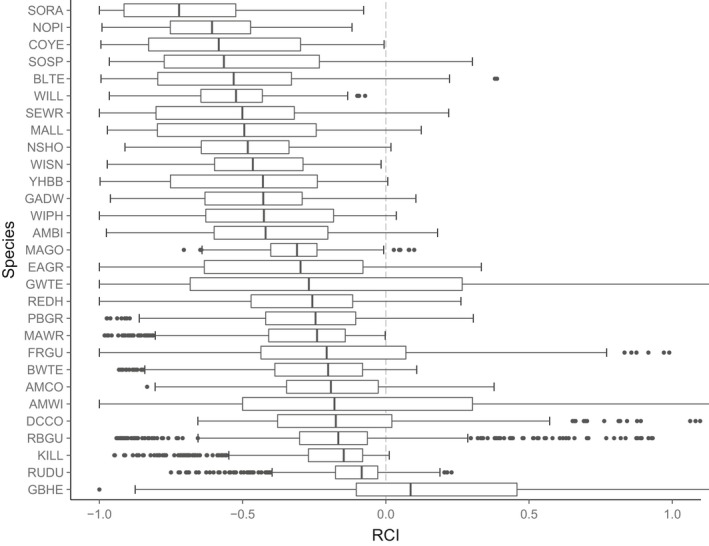
Variation in projected range change (range change index; RCI) to mid‐century for 29 wetland‐dependent bird species based on 1,080 projections per species. Boxplots show the median, and first and third quartiles, with whiskers showing the 1.5 interquartile range. Variation stems from choices of general circulation model (GCM), climate covariate hypothesis, threshold, and collinearity. See Table S1 for species abbreviations

**Figure 4:**
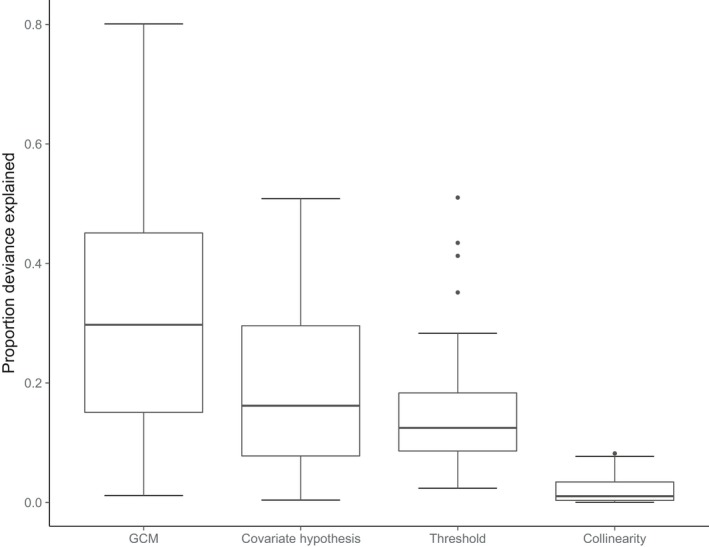
Proportion of deviance in range change index (RCI) explained by choice of: general circulation model (GCM), climate covariate hypothesis, collinearity, and threshold. Boxplots show the median, and first and third quartiles, with whiskers showing the 1.5 interquartile range

**Figure 5:**
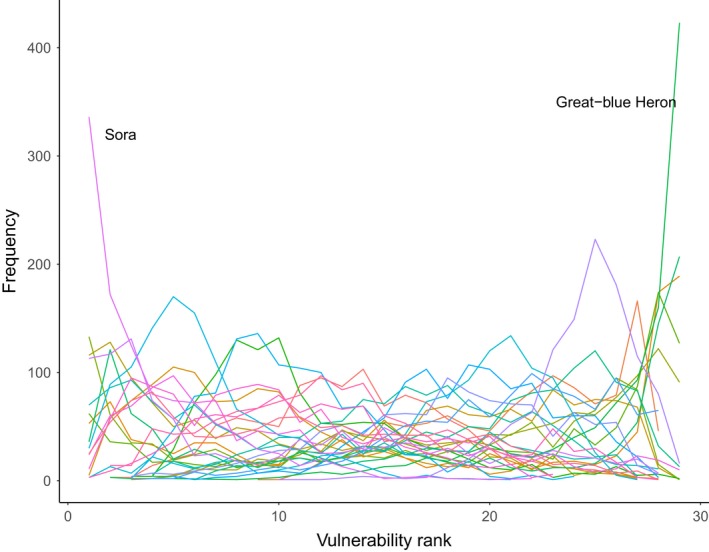
Frequency of vulnerability rankings for 29 wetland‐dependent bird species based on 1,080 projections per species. Rankings are based on the relative degree of projected range change (range change index; RCI). As expected, the two species with lowest and highest median RCI have relatively consistent vulnerability rankings

Range change estimates were highly variable. Although median values were negative for all but one species, the majority of projections included both negative and positive range change—that is, inferences to the direction and amount of range change depended critically on model‐building decisions (Figures 2 and 3). Note that this finding corresponds to the ensemble‐weighted projections; previous work has found similar divergence in projected effect for individual models (Araujo et al., 2005). GCM selection contributed the most uncertainty followed by the covariate hypothesis and thresholding procedure (Figure 4). The degree of collinearity retained in the covariate set was a minor source of uncertainty. Vulnerability rankings based on range change estimates were highly variable for most species meaning that modeling decisions qualitatively affect which species are identified as most vulnerable (Figure 5).

### 3.2 Effects of modeling decisions on amount of projected range change

Modeling decisions were associated with consistently more or less range change. The GCM that predicted the least range change (median RCI = −0.16) projected the least warming and the largest precipitation increase (# 34, Figs. S2 and S3 in Supporting Information). The GCM that projected the greatest range change (median RCI = −0.55) had near‐zero change in precipitation and the largest temperature increase (# 1). The use of bioclimatic and temporal climate covariates resulted in moderately more projected range loss compared to hydrological covariates (Table 1). Bioclimatic covariates projected 94%, and temporal covariates 98% more median range loss than hydrological covariates (Fig. S4 in Supporting Information). The difference projected between bioclimatic and temporal covariates was not statistically significant (Fig. S5 in Supporting Information). The impact of varying collinearity was low with the highest degree of collinearity projecting 9% more median range loss relative to the lowest (Table 1 and Fig. S6 in Supporting Information). The impact of using the fixed (0.5) threshold was very high compared with those that produced the least change (observed prevalence and averaged predicted probability) and resulted in projections of 94% more median range loss (Table 1 and Fig. S7 in Supporting Information). Numerous other thresholds resulted in moderate or low impacts to the amount of projected range loss (Table 1).

**Table 1:** Effects of different decisions on projections of range loss (using Range Change Index; RCI) and model performance in extrapolation tests. Interpretations presented in this table are based on model coefficients and confidence intervals (Figs. S5 and S8). For the RCI model, reference levels were those that predicted the least loss, and we report how much more loss was predicted by the alternative choices. For the model performance results, reference levels were those intermediate in performance. “Tendency” means that most, but not all, performance metrics indicate this result. For thresholding decisions, model performance is divided into locational accuracy and prevalence accuracy because results generally differed by these two sets of metrics. Locational accuracy is represented only by TSS because kappa generally did not differentiate among thresholding procedures in extrapolation, and AUC is not based on unique thresholds. Prevalence accuracy is represented by the prevalence match metric

Decision group	Decision	Effects on increased range loss	Effects on model performance in extrapolation	
Climate covariate hypothesis	Hydrological	None (*reference*)	Intermediate (tendency; *reference*)	
	Temporal	Moderate	Positive (tendency)	
	Bioclimatic	Moderate	Negative (tendency)	
Collinearity	NA	Low impact of *increasing* collinearity	Benefit to moderate (ten VIF) or higher collinearity (tendency)	
**Thresholding procedure**			**Locational accuracy**	**Prevalence accuracy**
	ObsPrev	None (reference)	Positive	Negative
	AvgProb	None	Positive	Negative
	PRplot based	Low	Intermediate	Intermediate
	ROC	Low	Positive	Intermediate
	SeSpeql	Low	Positive	Intermediate (reference)
	TSS	Low	Positive	Negative
	Fmeasure	Moderate	Intermediate (reference)	Positive
	Kappa	Moderate	Intermediate	Positive
	MidptProb	Moderate	Negative	Positive
	OPS	Moderate	Intermediate	Positive
	PredPrevObs	Moderate	Negative	Positive
	Fixed (0.5)	Very High	Negative	Intermediate

### 3.3 Effects of modeling decisions on model performance when extrapolating

Extrapolation to drought conditions was variously improved or diminished by different modeling decisions (Table 1). Compared to the hydrological hypothesis, the temporal hypothesis generally had a positive impact on extrapolation ability, while the bioclimatic hypothesis generally had a negative impact. Higher collinearity benefited extrapolations. For locational accuracy and prevalence accuracy, five thresholding procedures improved and three diminished projections compared with the reference levels of Fmeasure and SeSpeql, respectively.

All correlation coefficients between predictions of extrapolation performance based on cross‐validation performance and actual extrapolation performance were positive indicating cross‐validation performance measures provide value for making modeling decisions for extrapolating under climate change. Although all positive, they varied in strength, with ρ = 0.93 for AUC, ρ = 0.91 for TSS, ρ = 0.86 for kappa, and ρ = 0.72 for prevalence match.

## 4 DISCUSSION

Methodological decisions in projecting impacts of climate change can lead to widely divergent projections but are often made without strong justification. We found that future climatic conditions (i.e., GCM) were the largest source of uncertainty, but considerable variation in projected changes in range size also arose from the climate covariate hypothesis and the thresholding procedure. Projections based on hydrological covariates suggested reduced vulnerability compared to bioclimatic or temporal covariates. For reliably projecting climate change impacts, the simplest climate covariates may be the best: temporal covariates outperformed bioclimatic covariates and, by a slimmer margin, hydrological covariates. Cross‐validation performance was correlated with extrapolation performance; thus, our results support using cross‐validation performance to guide modeling decisions in climate change impacts studies. However, we note that methodological decisions profoundly affected which species were identified as most vulnerable (Figure 5). This finding implies that sensitivity analyses should remain a key part of any study aiming to inform management prioritization decisions (Wright et al., 2015).

### 4.1 Attributing uncertainty in range change projections

Whereas most other studies found various methodological uncertainties to be larger than the uncertainty in climate change itself (Buisson et al., 2010; Dormann, Purschke, Marquez, Lautenbach, & Schroder, 2008; Synes & Osborne, 2011), our study lends evidence that the plausible range of future climate itself is the largest unknown (see also Stralberg et al., 2015; Wenger et al., 2013). There is little agreement on metrics to separate “good” or “bad” GCMs (Knutti, Furrer, Tebaldi, Cermak, & Meehl, 2010); thus, we followed precedence and recommendations to represent the range of variation across GCMs (e.g., Fisichelli et al., 2016; Leppi, Rinella, Wilson, & Loya, 2014; Sofaer et al., 2016).

We observed substantial variation in projected future distributions depending on which covariate hypothesis was being tested. The underlying climate data were the same for all climate datasets, eliminating data source as the explanation for this result. Thus, variation in range size arose from the hypothesized relationship between climate and species distribution, and the particular derivations of climatic variation that represented each hypothesis. Collectively, our work and others (Pliscoff et al., 2014; Synes & Osborne, 2011) emphasize that sensitivity to climatic covariate sets apply across geographic regions and taxonomic groups.

We filtered our covariate sets using three different degrees of collinearity and found relatively little variation in range change projections. While posited as a serious concern with conflicting recommendations for best practices when projecting species distributions (Beaumont et al., 2005; Braunisch et al., 2013; Dormann et al., 2013), we found the impact of this decision to be relatively small. This may reflect our approach which started with a relatively large number of covariates (x̅ = 14) and ended in a moderate number (*x̅* = 9), thus potentially maintaining a lot of redundancy in covariate information, as in Beaumont et al. (2005).

We found that thresholding probabilities of occurrence into presence–absence can generate almost as much uncertainty as the climate hypothesis used. Serious concerns about the uncertainty stemming from choosing a thresholding procedure have been raised for predictions to current conditions (Freeman & Moisen, 2008; Jimenez‐Valverde & Lobo, 2007; Liu, Berry, Dawson, & Pearson, 2005), so it is not surprising that this contributes substantial uncertainty for future projections as Nenzen and Araujo (2011) also found.

### 4.2 Effects of modeling decisions on amount of projected range change

We projected range change under GCMs that projected wetter futures as well as those that projected drier overall futures. Our results suggest that increasing precipitation can compensate for range loss under modest temperature increases, but large increases in only temperature produced high projected range loss.

Because the temporal and bioclimatic hypotheses were likely more similar based on how they were computed—based on summaries and derivations of monthly precipitation and temperature data, versus an additional hydrological model—perhaps it is not surprising that they produced similar levels of range change. However, predictions of twice the range loss of the hydrological covariates are compelling. Schlaepfer, Lauenroth, & Bradford, (2012) also found more hopeful outcomes in projections using hydrological covariates versus more basic climatic covariates for future plant distributions. One likely explanation for our result is that temperature increases can be tempered by corresponding precipitation increases for ecosystems and processes that are water limited as in our study system, and this effect would be captured by water balance formulas of a hydrological model.

Whereas including more covariates generally produces more restricted predictions of ranges (e.g., Beaumont et al., 2005), the impacts of collinearity in projections of range change—where the measure is a difference in proportions of future and current ranges—have rarely been addressed. In our analysis, higher collinearity led to increases in range loss, consistent with the expectation that higher collinearity leads to overfit models that are not generalizable to new times or places (Heikkinen, Marmion, & Luoto, 2012).

The effects of thresholding methods on projected climate change vulnerability have received slim attention (Nenzen & Araujo, 2011). We found the fixed threshold of 0.5 produced alarming projections of range loss, nearly twice that of observed prevalence and average predicted probability procedures, which produced the most moderate projections of range loss. The fixed 0.5 threshold is known to overestimate occurrences of common species and underestimate occurrences of rare species (Freeman & Moisen, 2008; Jimenez‐Valverde & Lobo, 2007), and this bias may cause the fixed threshold to produce extreme estimates of range change for species that deviate from moderate levels of prevalence as ours do.

### 4.3 Effects of modeling decisions on model performance when extrapolating

Bioclimatic covariates have a strong theoretical link to niche modeling, are widely used for modeling climate change impacts, and are assumed to have numerous advantages (Austin, 2002; Booth et al., 2014; Watling et al., 2012); however, they underperformed in extrapolation relative to temporal covariates. The simpler summaries of temporal variation in temperature and precipitation could potentially represent more variability in climate if more months are represented in the ultimate set. The bioclimatic set had fewer covariates, but our result aligns with that of Peterson and Nakazawa (2008), who also found that bioclimatic covariates underperformed relative to other climatic covariates. They suggested that the indirect methods used to estimate the bioclimatic covariates relative to more direct use of means and summaries of other climate covariates may put bioclimatic variables at a disadvantage. Bioclimatic covariates representing seasonal extremes such as the “wettest quarter” may lead to errors in projections if the seasonality of future climate changes for species such as migratory birds with limited abilities to shift their phenology.

While hydrological variables have the advantage of representing water balance, in extrapolation—although better than bioclimatic covariates—they underperformed relative to temporal covariates. The noise introduced by the additional model required to derive these covariates could conceivably diminish the theorized benefit to these covariates, and this trade‐off should be explored further.

Recommended thresholding procedures varied depending on whether locational accuracy or prevalence accuracy was desired, and our results for extrapolation generally corroborated previous work assessing performance of thresholds under a static climate. For locational accuracy, like Liu et al. (2005), we found that sensitivity‐ and specificity‐based approaches (ROC, SeSpeql, TSS) as well as average probability and observed prevalence offered improved performance while the fixed threshold produced the poorest performance. For prevalence accuracy, like Freeman and Moisen (2008), we found kappa and predicted prevalence equals observed prevalence improved this metric, and our results additionally recommend Fmeasure, midpoint probability, and overall prediction success.

Positive correlation values between model performance in cross‐validation and extrapolation for all performance metrics (AUC, kappa, prevalence match, and TSS) indicate that cross‐validation performance can be used to select best choices for bioclimatic species distribution modeling. However, the smaller value for prevalence match indicates lower reliability of this metric, or, alternatively, the challenge of extrapolating prevalence. Conversely, the relatively high correlation values for AUC and TSS indicate these metrics may be among the better for making modeling decisions.

## 5 CONCLUSION

Climate change projections of range change should be guided by ecological knowledge of the factors limiting species distributions, whether via physiological or demographic mechanisms or via habitat availability. These considerations can guide the selection of climate covariates, which should be projected under a range of plausible futures. For mid‐century projections, GCM uncertainty represents much of the climate uncertainty, but for late‐century projections, it is necessary to include multiple emissions trajectories as well (Snover et al., 2013). Our results indicate that the thresholding procedure should be carefully justified or multiple methods considered, while collinearity has a relatively small effect. For decisions where ecological knowledge provides little guidance, our results support the use of cross‐validation to select methods that may then be applied under novel conditions. Visualizing and communicating the uncertainty arising from divergent climate futures and from modeling methods provide managers and other end users with critical context for decision‐making; sensitivity analyses will remain critical because variation arising from modeling decisions can lead to large uncertainties regarding the relative vulnerability of different species (Figure 5).

## AUTHOR CONTRIBUTIONS

Valerie Steen conceptualized, designed, executed, and interpreted the research. Barry Noon conceptualized and interpreted the research. Helen Sofaer conceptualized and interpreted the research. Susan Skagen conceptualized the research. Andrea Ray interpreted the research.

## CONFLICT OF INTEREST

None declared.
