# Supplementary material for: Projecting species’ vulnerability to climate change: Which uncertainty sources matter most and extrapolate best?
Source: Ecol Evol. 2017 Sep 20;7(21):8841–51. doi: 10.1002/ece3.3403 (PMC5677485; doi:10.1002/ece3.3403)
Supplement: Supplementary file 1 [file ECE3-7-8841-s001.docx]

Appendix S1: Additional methods and Tables S1-S2.

Prairie Pothole Ecosystem

Land cover in the Prairie Pothole Region (PPR) is a mosaic of grassland, cropland, and pothole wetlands. Pothole wetlands hold water on a permanent, semipermanent, seasonal, or temporary basis and, in some areas, reach densities greater than 40 km^-^² ([Kantrud *et al.*, 1989](#_ENREF_15)). The region is characterized by high climatic variability, and periods of drought or excessive precipitation may extend over multi-year periods. Historic multi-year droughts include the 10-year “dust bowl” drought in the 1930s and a 5-year drought from 1988-1992. During these pronounced drought periods, even many of the larger wetlands became dry ([Shjeflo, 1968](#_ENREF_27); [Winter & Rosenberry, 1998](#_ENREF_37); [Johnson *et al.*, 2004](#_ENREF_13)). Multi-year wet periods, or deluges, also occur and produce contrasting conditions with most landscape depressions holding surface water ([Beeri & Phillips, 2007](#_ENREF_5); [Niemuth *et al.*, 2010](#_ENREF_25)). The juxtaposition of wet and dry periods promote dramatic annual changes in marsh vegetative cover and high productivity ([Euliss *et al.*, 1999](#_ENREF_7); [van der Valk, 2005](#_ENREF_34); [Johnson *et al.*, 2010](#_ENREF_14)). Under sustained wet or dry conditions, marshes with high water levels have little emergent vegetation and those with low water levels support dense vegetative cover ([van der Valk & Davis, 1978](#_ENREF_35); [Johnson *et al.*, 2004](#_ENREF_13)).

Annual wetland-dependent bird counts are strongly positively correlated with that year’s wetland densities, demonstrating an immediate response to recent climatic conditions ([Stewart & Kantrud, 1973](#_ENREF_32); [Niemuth & Solberg, 2003](#_ENREF_24)). The amount and type of vegetative cover and wetland size are additional climate-mediated conditions that influence the distribution of wetland-dependent bird species in the PPR ([Weller & Spatcher, 1965](#_ENREF_36); [Murkin *et al.*, 1997](#_ENREF_22); [Johnson *et al.*, 2010](#_ENREF_14); [Steen & Powell, 2012](#_ENREF_31)).

Study Area

Our study area within the PPR was approximately 290,000 km² in size and included portions of three U.S. states: North Dakota, South Dakota, and Minnesota (Fig. S1). During our study period, mean annual temperature ranged from 3°C to 9°C from north to south and mean annual precipitation ranged from 300mm to 800mm west to east ([Millett *et al.*, 2009](#_ENREF_21)). Many wetlands in the PPR have been converted to cropland with wetland losses greatest in the eastern portion of our study area ([Dahl, 1990](#_ENREF_6); [Johnson *et al.*, 2008](#_ENREF_12)). Losses of surrounding grassland habitats have a similar geographic pattern (greatest in the eastern portion of the PPR) and are more extensive than wetland losses.

Breeding Bird Survey Data

Breeding bird Survey (BBS) routes are located on secondary roads throughout the U.S. and southern Canada. Morning surveys are conducted once annually during June. Route locations generally remain the same from year to year, although not all routes are surveyed each year. BBS routes consist of 50 survey stops spaced 0.8 km apart for a total length of 39.4 km. At each stop observers record all birds detected within 400 m for three minutes. Data are summarized as 10-stop totals by species (1/5 section of a route; <https://www.pwrc.usgs.gov/bbs/>). We used results from high-quality surveys (BBS class “run type 1”) for the years 1971-2010 derived from 72 routes within our study area: the PPR of North Dakota, South Dakota, and Minnesota - an area of 290,000 km² (Fig. S1). Because hydrological data were not comprehensive for 2000-2010 in Minnesota, 19 (of 24) Minnesota routes included surveys only through 1999. From previous work, we used consistently either the first or third section for a species depending on which section had higher detections for that species across all surveys due to temporal dependence of detections ([Steen *et al.*, 2014](#_ENREF_30)). We identified our set of focal species based on their prevalence (occurrence rate >5%; Table S1). To exclude the effects of temporal autocorrelation in occurrence patterns, we excluded consecutive years of survey data.

Land Cover Data

We extracted land cover variables to associate with BBS routes from GIS raster layers created by the U.S. Fish and Wildlife Service (USFWS; USFWS Regions 6 and 3 Habitat and Population Evaluation Teams, unpublished data). The USFWS data layers were at a 30-m resolution and a combined product based on classified Landsat Thematic Mapper Satellite imagery from 2000-2003 and USFWS National Wetlands Inventory (NWI) wetland polygons based on aerial imagery from the late 1970’s and early 1980’s. Classification accuracy of the upland land cover data for North and South Dakota, assessed in 2007, was > 90% (M. Estey, personal communication). Temporary, seasonal, and semipermanent wetlands are palustrine wetland classes describing the typical period they hold water, e.g. from a few weeks for a temporary wetland to multiple years for a semipermanent wetland. Water is a label applied to locations where water pixels in the newer imagery extended beyond NWI wetlands. Cropland included areas planted with crops or fallowed. Grassland included native prairie, planted grasslands, and hayland. Developed land cover included towns and residential areas. Tree habitat included small sections or rows of trees and, occasionally, areas of forest. To describe land cover surrounding survey route segments, we calculated the composition of each land cover type within a buffer of 0.4-km. We chose 0.4-km because it matched the maximum bird survey distance from the BBS survey route and because land cover covariates based on different buffer sizes were highly correlated. Land cover composition calculations were performed in Arcmap 10.0. We characterized wetlands into six classes: temporary, seasonal, semipermanent, lake, river, and water. We described upland habitat using four classes: cropland, grassland, tree, and developed. Land cover was assumed static across the survey interval (1971-2010) and for future projections.

Observed and Projected Climate Data

Both simulated past climate (“hindcast”) and projections of future climate (temperature, precipitation) were obtained from the "Downscaled CMIP3 and CMIP5 Climate and Hydrology Projections" archive (Bureau of Reclamation, 2014). This dataset provides Coupled Model Intercomparison Project Phase 5 (CMIP5) General Circulation Model (GCMs) data downscaled to 1/8° spatial resolution using the BCSD (Bias-Corrected Spatially Disaggregated) approach ([Wood *et al.*, 2004](#_ENREF_38); [Maurer *et al.*, 2007](#_ENREF_20)). In our study area, 1/8° represents an east to west spacing of approximately 10 km and north to south spacing of approximately 14 km. This downscaled temperature and precipitation data also had been processed through the Variable Infiltration Capacity (VIC) macroscale hydrologic model ([version 4.1.2h, Liang, 1994](#_ENREF_17)), to obtain projections of hydrological variables.

We used the output from 10 randomly selected CMIP5 GCMs (represented as circled numbers in Fig. S2). These GCMs well-represent the range of plausible futures in the GCMs, from less warming (+~ 1.5 °C) to greater warming (over +4 °C) and from a decrease of ~7% in annual precipitation to an increase of ~20%. Whereas only one GCM projected a notable decrease in precipitation, the risk of less precipitation coupled with the higher temperature increase in that GCM (+ ~4 °C; #27, Fig. S2) is an important risk to consider for the Prairie Pothole Region and an important risk to include in the analysis. We chose only GCMs run with the RCP 8.5 greenhouse gas emissions pathway for our mid-century projections ([Snover *et al.*, 2013](#_ENREF_28)).

Candidate Climate Covariates

The candidate covariates in our temporal hypothesis included seasonal, yearly, and multi-year summaries as well as temporal variation (standard deviation) because research in the PPR has shown the influence of shorter and longer term climate as well as the variability in driving wetland habitats ([Larson, 1995](#_ENREF_16); [Johnson *et al.*, 2010](#_ENREF_14)). For the bioclimatic hypothesis, we defined candidate bioclimatic covariates to describe avian distributions from Synes and Osborne ([2011](#_ENREF_33)) and Jimenez-Valverde et al. ([2011](#_ENREF_11)). Growing degree days and moisture index were calculated according to Synes and Osborne ([2011](#_ENREF_33)) with the exception that potential evapotranspiration in the moisture index formula was output from the VIC hydrological model which used the Penman-Monteith equation. The candidate covariates in our hydrological hypothesis included those that predict yearly density of wetlands holding water in the PPR ([Sofaer *et al.*, 2016](#_ENREF_29)) as well as additional hydrological covariates representing seasonal wetness patterns expected to change in the future ([Ballard *et al.*, 2014](#_ENREF_3)) and late spring and early summer wetness patterns that may affect settling patterns of migratory birds ([Heikkinen *et al.*, 2006](#_ENREF_10)). The hydrological hypothesis included covariates from the VIC hydrological model and derivations of temperature and precipitation.

Species Distribution Models

For each species’ SDM, we used the species’ yearly occurrence – defined as ≥ 1 detection per survey section – as the response variable and climate and land cover covariates as predictor variables. Climate covariates assigned to a given BBS route were based on the nearest gridded climate data point and were temporally matched to the year of the BBS survey, an uncommon approach that has improved models for species sensitive to annual climate variability (but see, e.g., [Reside *et al.*, 2010](#_ENREF_26); [Bateman *et al.*, 2016](#_ENREF_4)).

In the Biomod package, we employed seven modeling algorithms to fit covariates to species occurrence data. These included generalized linear models (GLM) with polynomial terms and without model selection, generalized boosted models (GBM), random forests (RF; with 2500 trees), multivariate adaptive regression splines (MARS), artificial neural networks (ANN), classification tree analysis (CTA), and flexible discriminant analysis (FDA). For additional information on settings of models, see the default settings for Biomod2. Because consensus probabilities are expected to perform better than probabilities based on a single modeling technique, we used the consensus of the probability of occurrence as our prediction ([Araujo *et al.*, 2005](#_ENREF_2); [Marmion *et al.*, 2009](#_ENREF_19); [Garcia *et al.*, 2012](#_ENREF_9)). Consensus was estimated from the weighted mean probability of occurrence across those modeling algorithms that achieved a predictive performance of AUC ≥ 0.65. The weights were based on the AUC values for each model. Focal species were removed when no model algorithm achieved a minimum AUC of 0.65. Twenty-nine wetland-dependent bird species met the criteria for inclusion (Table S2).

Thresholding Procedures

We evaluated 12 of 14 probability of occurrence thresholding procedures (Table S3) assessed by Nenzen and Araujo ([2011](#_ENREF_23)). We did not include precision-recall minimized (PRmin) and maximize sum of sensitivity and specificity (SeSpmax) because we found they were highly similar to or the same as predicted prevalence equals observed prevalence (PredPrev=ObsPrev) and the true skill statistic (TSS), respectively.

Range Change Index

Range Change Index (RCI) is the number of pixels gained minus the number of pixels lost divided by the number of pixels currently occupied. It compares the size of the projected and current distributions for species with unlimited dispersal capabilities as expected for vagile bird species; it does not assess spatial shifts. For each GCM, pixels gained were based on the GCM forecast, and pixels lost were based on the GCM hindcast. Models for predicting RCI were trained with the full training dataset (Table S1).

Influential species

Influential species in one or more GLMM included Great-blue Heron (*Ardea herodias*), Green-winged Teal (*Anas crecca*), American Wigeon (*Anas americana*), Franklin’s Gull (*Leucophaeus pipixcan*), Sedge Wren (*Cistothorus platensis*), Common Yellowthroat (*Geothlypis trichas*), Killdeer (*Charadrius vociferus*), Eared Grebe (*Podiceps nigricollis*), and Yellow-headed Blackbird (*Xanthocephalus xanthocephalus*).

Model performance metrics

Cohen’s kappa statistic (kappa) corrects overall prediction success by the expected correct predictions occurring by chance ([Manel *et al.*, 2001](#_ENREF_18)). True Skill Statistic (TSS) essentially measure the sum of sensitivity and specificity and is unaffected by prevalence ([Allouche *et al.*, 2006](#_ENREF_1)). Area under the receiver operating characteristic curve (AUC) is based on a plot of sensitivity versus 1-specificity across all thresholds ([Fielding & Bell, 1997](#_ENREF_8)). Prevalence match was defined as 1 minus the absolute value of the difference between predicted and actual prevalence.

Table S1. Species, species alpha code, and prevalence (proportion of surveys with species present) for each dataset.

|  |  |  | Prevalence | | |
| --- | --- | --- | --- | --- | --- |
| Common Name | Scientific Name | Alpha Code | Training  (all years; n=854) | Wet & Normal years (n=702) | Dry years (n=152) |
| Gadwall | *Anas strepera* | GADW | 0.28 | 0.28 | 0.26 |
| American Wigeon | *Anas americana* | AMWI | 0.06 | 0.06 | 0.04 |
| Mallard | *Anas platyrhynchos* | MALL | 0.6 | 0.6 | 0.58 |
| Blue-winged Teal | *Anas discors* | BWTE | 0.39 | 0.42 | 0.3 |
| Northern Shoveler | *Anas clypeata* | NSHO | 0.18 | 0.19 | 0.14 |
| Northern Pintail | *Anas acuta* | NOPI | 0.24 | 0.26 | 0.16 |
| Green-winged Teal | *Anas crecca* | GWTE | 0.06 | 0.06 | 0.05 |
| Redhead | *Aythya americana* | REDH | 0.13 | 0.14 | 0.11 |
| Ruddy Duck | *Oxyura jamaicensis* | RUDU | 0.13 | 0.14 | 0.11 |
| Eared Grebe | *Podiceps nigricollis* | EAGR | 0.04 | 0.04 | 0.05 |
| Pied-billed Grebe | *Podilymbus podiceps* | PBGR | 0.23 | 0.26 | 0.11 |
| Double-crested Cormorant | *Phalacrocorax auritus* | DCCO | 0.10 | 0.09 | 0.11 |
| American Bittern | *Botaurus lentiginosus* | AMBI | 0.23 | 0.25 | 0.15 |
| Great Blue Heron | *Ardea herodias* | GBHE | 0.06 | 0.06 | 0.05 |
| Sora | *Porzana carolina* | SORA | 0.27 | 0.30 | 0.15 |
| American Coot | *Fulica americana* | AMCO | 0.29 | 0.32 | 0.12 |
| Killdeer | *Charadrius vociferus* | KILL | 0.83 | 0.84 | 0.78 |
| Willet | *Tringa semipalmata* | WILL | 0.17 | 0.17 | 0.16 |
| Marbled Godwit | *Limosa fedoa* | MAGO | 0.20 | 0.20 | 0.23 |
| Wilson’s Snipe | *Gallinago delicata* | WISN | 0.19 | 0.19 | 0.16 |
| Wilson’s Phalarope | *Phalaropus tricolor* | WIPH | 0.11 | 0.12 | 0.11 |
| Franklin’s Gull | *Leucophaeus pipixcan* | FRGU | 0.12 | 0.12 | 0.08 |
| Ring-billed Gull | *Larus delawarensis* | RBGU | 0.14 | 0.14 | 0.14 |
| Black Tern | *Chlidonias niger* | BLTE | 0.19 | 0.21 | 0.12 |
| Sedge Wren | *Cistothorus platensis* | SEWR | 0.24 | 0.25 | 0.22 |
| Marsh Wren | *Cistothorus palustris* | MAWR | 0.23 | 0.24 | 0.23 |
| Common Yellowthroat | *Geothlypis trichas* | COYE | 0.83 | 0.83 | 0.81 |
| Song Sparrow | *Melospiza melodia* | SOSP | 0.66 | 0.66 | 0.63 |
| Yellow-headed Blackbird | Xanthocephalus xanthocephalus | YHBL | 0.56 | 0.56 | 0.55 |

Table S2. Nine sets of climate covariates were used in species distribution models. Sets varied by three hypotheses (temporal, bioclimatic, and hydrological) and each hypothesis originally included all candidate covariates prior to variable reduction to reduce collinearity. Variables were eliminated based on their variance inflation factor (VIF) values relative to two thresholds: 10 and 2. Rows in the table are ordered according to type of variable (precipitation, hydrological/water balance, and temperature) and within these types are ordered from finest temporal scale to broadest (e.g. from month to 10-yr). Year was a biological year defined to match the annual cycle of migratory birds—that is, the 12 month period leading up to and including May - the typical month of breeding initiation for our group of species.

| **Covariate** | **Temporal** | | | **Bioclimatic** | | | **Hydrological** | | |
| --- | --- | --- | --- | --- | --- | --- | --- | --- | --- |
|  | All | VIF=10 | VIF=2 | All | VIF=10 | VIF=2 | All | VIF=10 | VIF=2 |
| Precip., driest month |  |  |  | X | X | X |  |  |  |
| Precip., wettest month |  |  |  | X | X | X |  |  |  |
| Precip., spring | X | X | X |  |  |  |  |  |  |
| Precip., winter | X | X | X |  |  |  |  |  |  |
| Precip., fall | X | X | X |  |  |  |  |  |  |
| Precip., summer | X | X | X |  |  |  |  |  |  |
| Precip., summer + September |  |  |  | X | X | X |  |  |  |
| Precip., current year |  |  |  | X |  |  |  |  |  |
| Precip., 5 year (inc. current yr) | X |  |  |  |  |  |  |  |  |
| Precip., 10 year (inc. current yr) | X | X |  |  |  |  |  |  |  |
| Precip. std. dev., 5 year | X | X | X |  |  |  |  |  |  |
| Precip., std. dev., 10 year | X | X | X |  |  |  |  |  |  |
| Soil moisture content, May |  |  |  |  |  |  | X |  |  |
| Soil moisture content, October |  |  |  |  |  |  | X | X | X |
| Runoff, April |  |  |  |  |  |  | X | X | X |
| Runoff, May |  |  |  |  |  |  | X | X | X |
| Soil moisture content, May – June |  |  |  |  |  |  | X | X | X |
| Precip. – PET, spring |  |  |  |  |  |  | X | X |  |
| Precip. – PET, winter |  |  |  |  |  |  | X | X | X |
| Precip. – PET, fall |  |  |  |  |  |  | X | X | X |
| Precip.- PET, summer |  |  |  |  |  |  | X | X | X |
| Precip.- PET, annual |  |  |  |  |  |  | X |  |  |
| PET, annual |  |  |  |  |  |  | X | X |  |
| Moisture Index |  |  |  | X | X |  |  |  |  |
| Precip.- PET, 5-yr with 1-yr lag |  |  |  |  |  |  | X | X |  |
| Growing degree days |  |  |  | X | X | X |  |  |  |
| Temp. range, first month with mean above freezing |  |  |  |  |  |  | X | X | X |
| Temp., max of max summer monthlies |  |  |  |  |  |  | X | X | X |
| Temp., coldest month |  |  |  | X | X | X |  |  |  |
| Temp., warmest month |  |  |  | X | X | X |  |  |  |
| Temp., mean of max spring monthlies |  |  |  |  |  |  | X | X | X |
| Temp., mean, coldest quarter |  |  |  | X | X |  |  |  |  |
| Temp., mean, warmest quarter |  |  |  | X | X |  |  |  |  |
| Temp., spring | X | X | X |  |  |  |  |  |  |
| Temp., winter | X | X |  |  |  |  |  |  |  |
| Temp., fall | X | X | X |  |  |  |  |  |  |
| Temp., summer | X | X | X |  |  |  |  |  |  |
| Temp., annual |  |  |  | X |  |  |  |  |  |
| Temp., 5 year | X |  |  |  |  |  |  |  |  |
| Temp., 10 year | X | X |  |  |  |  |  |  |  |
| Temp., std. dev., 5 year | X | X | X |  |  |  |  |  |  |
| Temp., std. dev., 10 year | X | X | X |  |  |  |  |  |  |

Table S3. Description of thresholding techniques ([these taken from Nenzen & Araujo, 2011](#_ENREF_23)).

| **Technique** | **Description** |
| --- | --- |
| AveProb | Mean probability of predicted presence |
| Fixed (0.5) | Traditional method of using 0.5 probability across models |
| Fmeasure | Maximize F=2*true positives/(presences + true positive + false positive) |
| Kappa | Maximize Cohen’s kappa statistic |
| MidptProb | Median of probabilities between presences and absences in the dataset |
| ObsPrev | Observed prevalence (proportion of presences) |
| OPS | Overall prediction success; sum of true positives and true negatives divided by the number of observations |
| PredPrevObs | Predicted prevalence matches observed prevalence |
| PRplotbased | Precision-recall plot; minimize distance to upper left corner of precision-recall plot |
| ROC | Minimize distance to the upper left corner of the receiver operator curve |
| SeSpeql | Minimize difference between sensitivity and specificity |
| TSS (True Skill Statistic) | Maximize sensitivity + specificity -1 |

**REFERENCES**

Allouche, O., Tsoar, A. & Kadmon, R. (2006) Assessing the accuracy of species distribution models: prevalence, kappa and the true skill statistic (TSS). *Journal of Applied Ecology*, **43**, 1223-1232.

Araujo, M.B., Whittaker, R.J., Ladle, R.J. & Erhard, M. (2005) Reducing uncertainty in projections of extinction risk from climate change. *Global Ecology and Biogeography*, **14**, 529-538.

Ballard, T., Seager, R., Smerdon, J.E., Cook, B.I., Ray, A.J., Rajagopalan, B., Kushnir, Y., Nakamura, J. & Henderson, N. (2014) Hydroclimate Variability and Change in the Prairie Pothole Region, the "Duck Factory'' of North America. *Earth Interactions*, **18**, 1-28.

Bateman, B.L., Pidgeon, A.M., Radeloff, V.C., VanDerWal, J., Thogmartin, W.E., Vavrus, S.J. & Heglund, P.J. (2016) The pace of past climate change vs. potential bird distributions and land use in the United States. *Global Change Biology*, **22**, 1130-1144.

Beeri, O. & Phillips, R.L. (2007) Tracking palustrine water seasonal and annual variability in agricultural wetland landscapes using Landsat from 1997 to 2005. *Global Change Biology*, **13**, 897-912.

Bureau of Reclamation (2014) *Downscaled CMIP3 and CMIP5 Climate and Hydrology Projections: Release of Downscaled CMIP5 Climate Projections, Comparison with preceding Information, and Summary of User Needs.* U.S. Department of the Interior, Bureau of Reclamation, Technical Services Center, Denver, Colorado. 110pp.

Dahl, T.E. (1990) *Wetlands losses in the United States 1780's to 1980's.* In: U.S. Department of the Interior, Fish and Wildlife Service, Washington, D.C.

Euliss, N.H., Wrubleski, D.A. & Mushet, D.M. (1999) Wetlands of the Prairie Pothole Region: invertebrate species composition, ecology, and management. *Invertebrates in Freshwater Wetlands of North America: Ecology and Management* (ed. by D.P. Batzer, Rader, R.B. And Wissinger, S.A.), pp. 471-514. John Wiley & Sons Inc, New York, USA.

Fielding, A.H. & Bell, J.F. (1997) A review of methods for the assessment of prediction errors in conservation presence/absence models. *Environmental Conservation*, **24**, 38-49.

Garcia, R.A., Burgess, N.D., Cabeza, M., Rahbek, C. & Araujo, M.B. (2012) Exploring consensus in 21st century projections of climatically suitable areas for African vertebrates. *Global Change Biology*, **18**, 1253-1269.

Heikkinen, R.K., Luoto, M. & Virkkala, R. (2006) Does seasonal fine-tuning of climatic variables improve the performance of bioclimatic envelope models for migratory birds? *Diversity and Distributions*, **12**, 502-510.

Jimenez-Valverde, A., Barve, N., Lira-Noriega, A., Maher, S.P., Nakazawa, Y., Papes, M., Soberon, J., Sukumaran, J. & Peterson, A.T. (2011) Dominant climate influences on North American bird distributions. *Global Ecology and Biogeography*, **20**, 114-118.

Johnson, R.R., Oslund, F.T. & Hertel, D.R. (2008) The past, present, and future of prairie potholes in the United States. *Journal of Soil and Water Conservation*, **63**, 84a-87a.

Johnson, W.C., Boettcher, S.E., Poiani, K.A. & Guntenspergen, G. (2004) Influence of weather extremes on the water levels of glaciated prairie wetlands. *Wetlands*, **24**, 385-398.

Johnson, W.C., Werner, B., Guntenspergen, G.R., Voldseth, R.A., Millett, B., Naugle, D.E., Tulbure, M., Carroll, R.W.H., Tracy, J. & Olawsky, C. (2010) Prairie Wetland Complexes as Landscape Functional Units in a Changing Climate. *Bioscience*, **60**, 128-140.

Kantrud, H.A., Krapu, G.L. & Swanson, G.A. (1989) Prairie basin wetlands of the Dakotas: a community profile. In: *Biological Report 85 (7. 28)*, p. 111. U.S. Fish and Wildlife Service, Washington, DC, USA.

Larson, D.L. (1995) Effects of Climate on Numbers of Northern Prairie Wetlands. *Climatic Change*, **30**, 169-180.

Liang, X. (1994) A two-layer variable infiltration capacity land surface representation for general circulation models.

Manel, S., Williams, H.C. & Ormerod, S.J. (2001) Evaluating presence-absence models in ecology: the need to account for prevalence. *Journal of Applied Ecology*, **38**, 921-931.

Marmion, M., Parviainen, M., Luoto, M., Heikkinen, R.K. & Thuiller, W. (2009) Evaluation of consensus methods in predictive species distribution modelling. *Diversity and Distributions*, **15**, 59-69.

Maurer, E.P., Brekke, L., Pruitt, T. & Duffy, P.B. (2007) Fine‐resolution climate projections enhance regional climate change impact studies. *Eos, Transactions American Geophysical Union*, **88**, 504-504.

Millett, B., Johnson, W.C. & Guntenspergen, G. (2009) Climate trends of the North American prairie pothole region 1906-2000. *Climatic Change*, **93**, 243-267.

Murkin, H.R., Murkin, E.J. & Ball, J.P. (1997) Avian habitat selection and prairie wetland dynamics: a 10-year experiment. *Ecological Applications*, **7**, 1144-1159.

Nenzen, H.K. & Araujo, M.B. (2011) Choice of threshold alters projections of species range shifts under climate change. *Ecological Modelling*, **222**, 3346-3354.

Niemuth, N.D. & Solberg, J.W. (2003) Response of waterbirds to number of wetlands in the Prairie Pothole Region of North Dakota, USA. *Waterbirds*, **26**, 233-238.

Niemuth, N.D., Wangler, B. & Reynolds, R.E. (2010) Spatial and Temporal Variation in Wet Area of Wetlands in the Prairie Pothole Region of North Dakota and South Dakota. *Wetlands*, **30**, 1053-1064.

Reside, A.E., VanDerWal, J.J., Kutt, A.S. & Perkins, G.C. (2010) Weather, Not Climate, Defines Distributions of Vagile Bird Species. *Plos One*, **5**, e13569.

Shjeflo, J.B. (1968) *Evapotranspiration and the water budget of prairie potholes in North Dakota*. U.S. Govt. Print. Off., Washington, D.C.

Snover, A.K., Mantua, N.J., Littell, J.S., Alexander, M.A., Mcclure, M.M. & Nye, J. (2013) Choosing and Using Climate-Change Scenarios for Ecological-Impact Assessments and Conservation Decisions. *Conservation Biology*, **27**, 1147-1157.

Sofaer, H.R., Skagen, S.K., Barsugli, J.J., Rashford, B.S., Reese, G.C., Hoeting, J.A., Wood, A.W. & Noon, B.R. (2016) Projected wetland densities under climate change: habitat loss but little geographic shift in conservation strategy. *Ecological Applications*, **26**, 1677-1692.

Steen, V., Skagen, S.K. & Noon, B.R. (2014) Vulnerability of Breeding Waterbirds to Climate Change in the Prairie Pothole Region, USA. *Plos One*, **9**, e96747.

Steen, V.A. & Powell, A.N. (2012) Wetland Selection by Breeding and Foraging Black Terns in the Prairie Pothole Region of the United States. *Condor*, **114**, 155-165.

Stewart, R.E. & Kantrud, H.A. (1973) Ecological Distribution of Breeding Waterfowl Populations in North-Dakota. *Journal of Wildlife Management*, **37**, 39-50.

Synes, N.W. & Osborne, P.E. (2011) Choice of predictor variables as a source of uncertainty in continental-scale species distribution modelling under climate change. *Global Ecology and Biogeography*, **20**, 904-914.

van der Valk, A.G. (2005) Water-level fluctuations in North American prairie wetlands. *Hydrobiologia*, **539**, 171-188.

van der Valk, A.G. & Davis, C.B. (1978) The role of seed banks in the vegetation dynamics of prairie glacial marshes. *Ecology*, **59**, 322-335.

Weller, M.W. & Spatcher, C.S. (1965) Role of habitat in the distribution and abundance of marsh birds. In: *Special Report no. 43*. Iowa Agriculture and Home Economics Experiment Station, Ames, Iowa.

Winter, T.C. & Rosenberry, D.O. (1998) Hydrology of prairie pothole wetlands during drought and deluge: a 17-year study of the Cottonwood Lake wetland complex in North Dakota in the perspective of longer term measured and proxy hydrological records. *Climatic Change*, **40**, 189-209.

Wood, A.W., Leung, L.R., Sridhar, V. & Lettenmaier, D.P. (2004) Hydrologic implications of dynamical and statistical approaches to downscaling climate model outputs. *Climatic Change*, **62**, 189-216.
