# Supplementary material for: Projecting species’ vulnerability to climate change: Which uncertainty sources matter most and extrapolate best?
Source: Ecol Evol. 2017 Sep 20;7(21):8841–51. doi: 10.1002/ece3.3403 (PMC5677485; doi:10.1002/ece3.3403)
Supplement: Supplementary file 2 [file ECE3-7-8841-s002.docx]

Appendix S2: Additional Figures (S1-S8).

Figure S1. Study area in the U.S. Prairie Pothole Region with 72 Breeding Bird Survey routes from which we obtained bird occurrence data.

Figure S2. Change in temperature and precipitation projected by 2041-2070 relative to 1971-2000 for bias-corrected GCMs under two greenhouse gas concentration trajectories (RCP 4.5 and RCP 8.5) in the U.S. Prairie Potholes in North Dakota, South Dakota, and Minnesota. The ten GCMs used in this study are circled. Each GCM is identified by a number: 1 = ACCESS3-0; 2 = ACCESS3-3; 3 = BCC-CSM1-1-M; 4 = BCC-CSM1-1, 5 = CanESM2, 6 = CCSM4, 7 = CESM1-BGC, 8 = CESM1-CAM5, 9 = CMCC-CM, 10 = CNRM-CM5, 11 = CSIRO-Mk3-6-0, 12 = EC-EARTH, 13 = FGOALS-g2, 14 = FGOALS-S4, 15 = FIO-ESM, 16 = GFDL-CM3, 17 = GFDL-ESM2G, 18 = GFDL-ESM2M, 19 = GISS-E2-H-CC, 20 = GISS-E2-R, 21 = GISS-E2-R-CC, 22 = HadGEM2-AO, 23 = HadGEM2-CC, 24 = HadGEM2-ES, 25 = INMCM4, 26 = IPSL-CM5A-LR, 27 = IPSL-CM5A-MR, 28 = IPSL-CM5B-LR, 29 = MIROC-ESM, 30 = MIROC-ESM-CHEM, 31 = MIROC5, 32 = MPI-ESM-LR, 33 = MPI-ESM-MR, 34 = MRI-CGCM3, 35 = NorESM1-M, 36 = NorESM1-ME. Data provided by Marian Talbert.

Figure S3. Variation in Range Change Index (RCI) by GCM. Boxplots show the median, and first and third quartiles, with whiskers showing the 1.5 inter-quartile range.

Figure S4. Variation in Range Change Index (RCI) by covariate hypothesis. Boxplots show the median, and first and third quartiles, with whiskers showing the 1.5 inter-quartile range.

Figure S5. Estimates of relationships between decisions for general circulation model (GCM), climate hypothesis, collinearity, thresholding procedure and range change index (RCI). Reference levels (thus, not shown in the plot) were chosen for each uncertainty source based on the level with the smallest negative relationship with RCI: ObsPrev (threshold), hydrological (climate hypothesis), and mri (GCM). The “Q” and “L” suffixes on collinearity denote quadratic and linear effects of higher collinearity. Thick portions of bars represent 90% confidence intervals with thin portions extending to 95% confidence intervals.

Figure S6. Variation in Range Change Index (RCI) by degree of covariate collinearity: all covariates; covariate sets reduced to a moderate variance inflation factor (VIF) of ten; and covariate sets reduced to a more stringent VIF of two. Boxplots show the median, and first and third quartiles, with whiskers showing the 1.5 inter-quartile range.

Figure S7. Variation in Range Change Index (RCI) by thresholding procedure. Boxplots show the median, and first and third quartiles, with whiskers showing the 1.5 inter-quartile range.

Figure S8. Estimates of relationships between decisions for general circulation model (GCM), climate hypothesis, collinearity, thresholding procedure and model performance for four model performance metrics: AUC, True Skill Statistic (TSS), kappa, and prevalence match. Cross-validation results reflect predictions to randomly subsetted data. Extrapolation results reflect predictions from models trained with normal and wet years data and evaluated on drought years. Not shown because they were used as reference levels in the model are: hydrological, Fmeasure (kappa and TSS models) and SeSpeql (prevalence match model). The “Q” and “L” suffixes on collinearity denote quadratic and linear effects of higher collinearity. Thick portions of bars represent 90% confidence intervals with thin portions extending to 95% confidence intervals.
