# Supplementary figures and images for: Projecting species’ vulnerability to climate change: Which uncertainty sources matter most and extrapolate best?
Source: Ecol Evol. 2017 Sep 20;7(21):8841–51. doi: 10.1002/ece3.3403 (PMC5677485; doi:10.1002/ece3.3403)

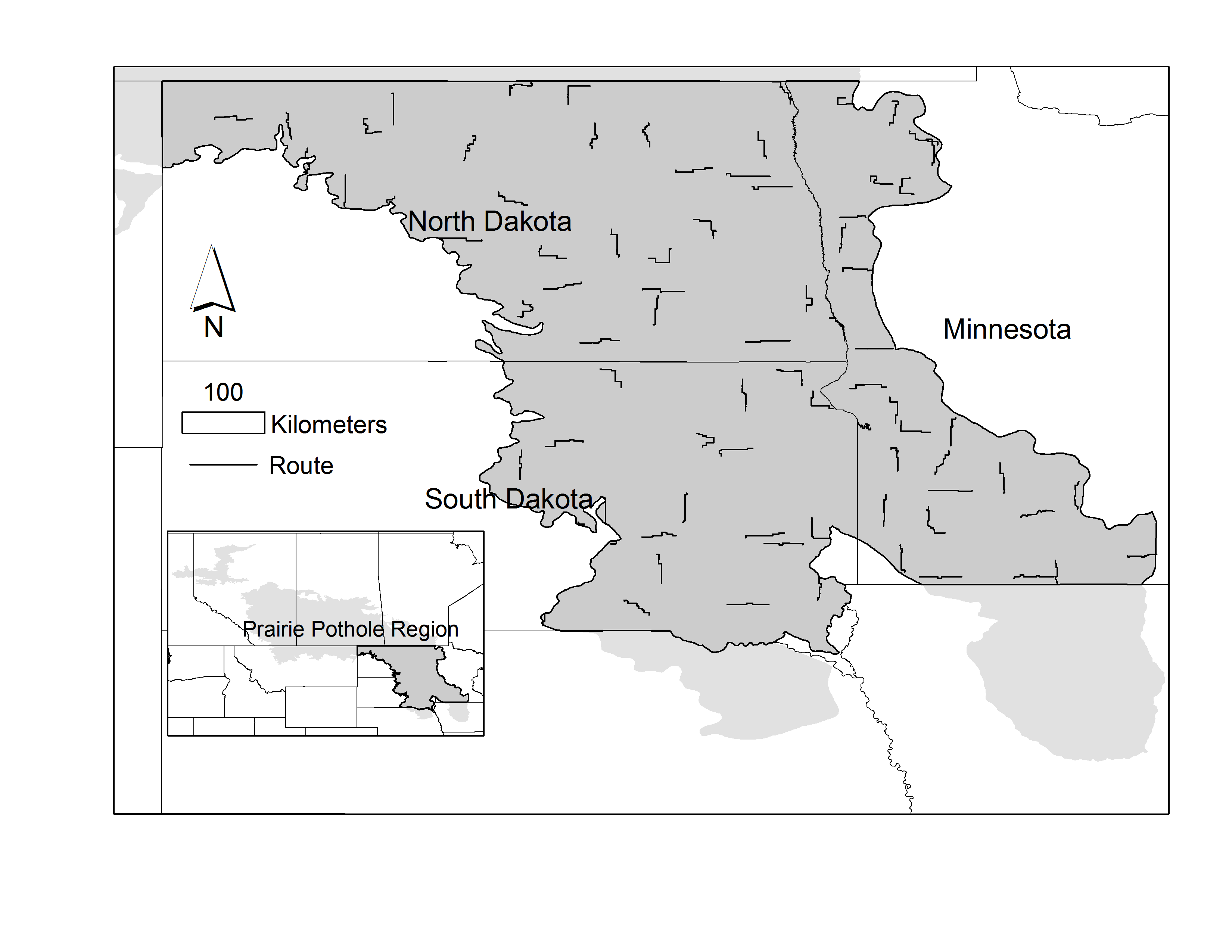

Supplement: Supplementary file 3 [file ECE3-7-8841-s003.tif]

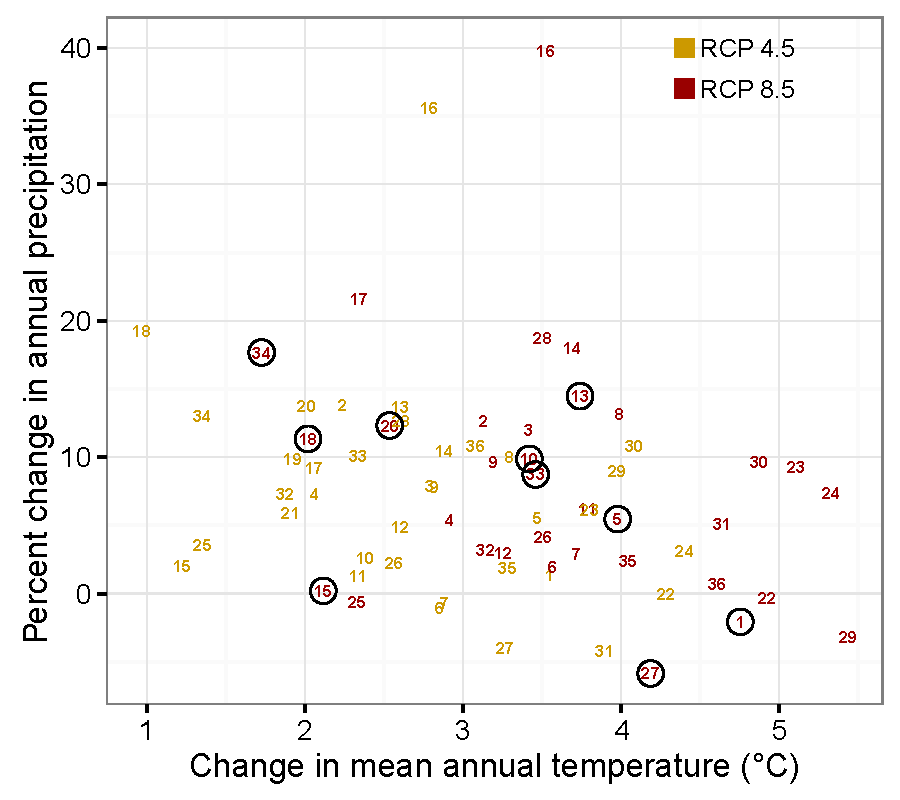

Supplement: Supplementary file 4 [file ECE3-7-8841-s004.tiff]

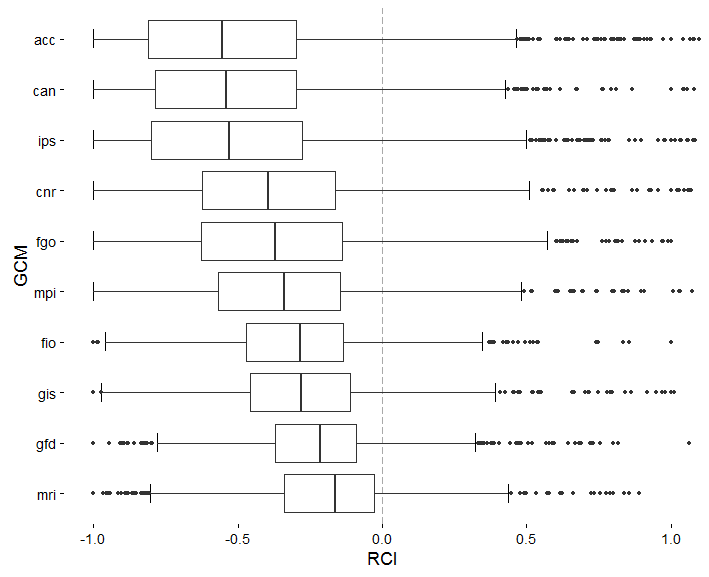

Supplement: Supplementary file 5 [file ECE3-7-8841-s005.tiff]

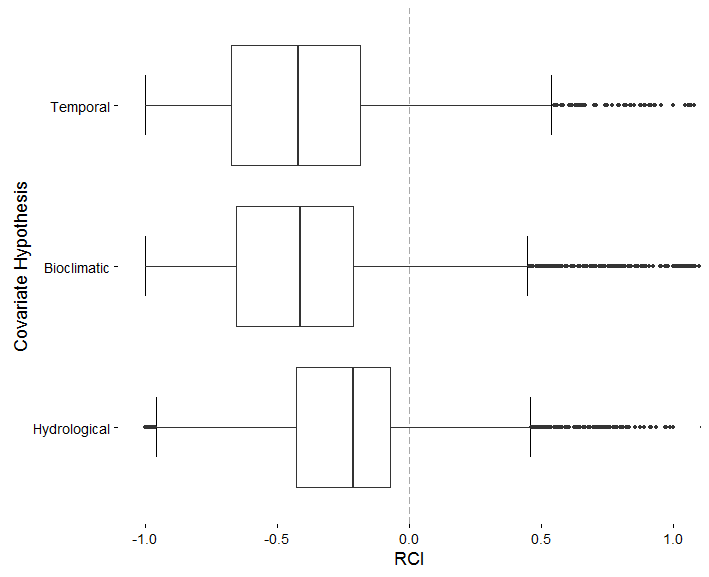

Supplement: Supplementary file 6 [file ECE3-7-8841-s006.tiff]

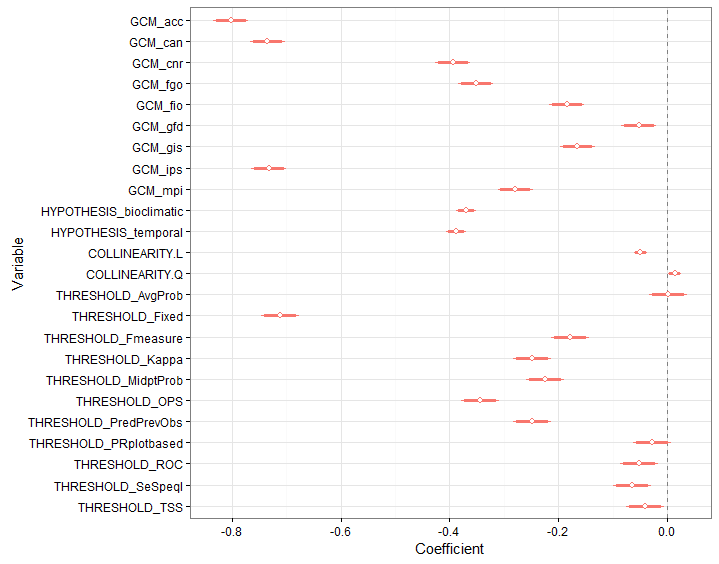

Supplement: Supplementary file 7 [file ECE3-7-8841-s007.tiff]

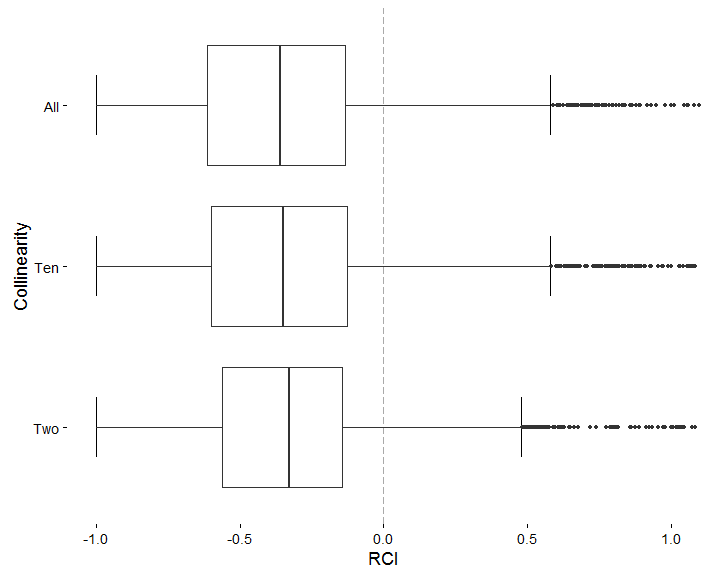

Supplement: Supplementary file 8 [file ECE3-7-8841-s008.tiff]

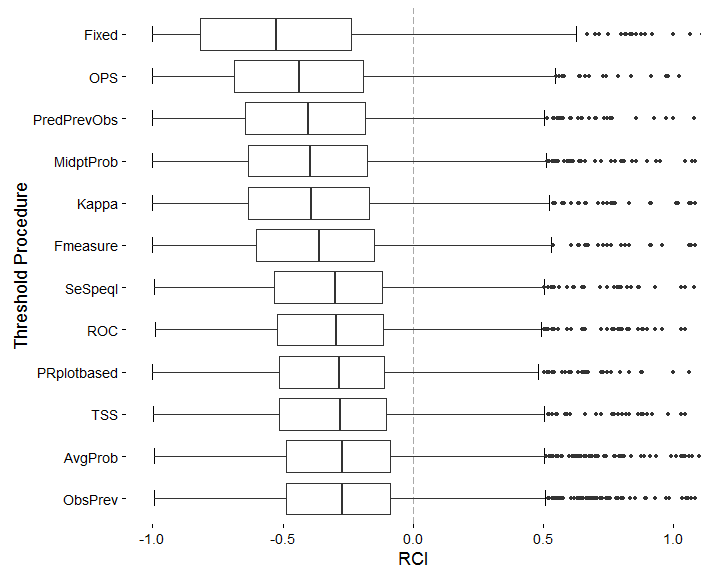

Supplement: Supplementary file 9 [file ECE3-7-8841-s009.tiff]

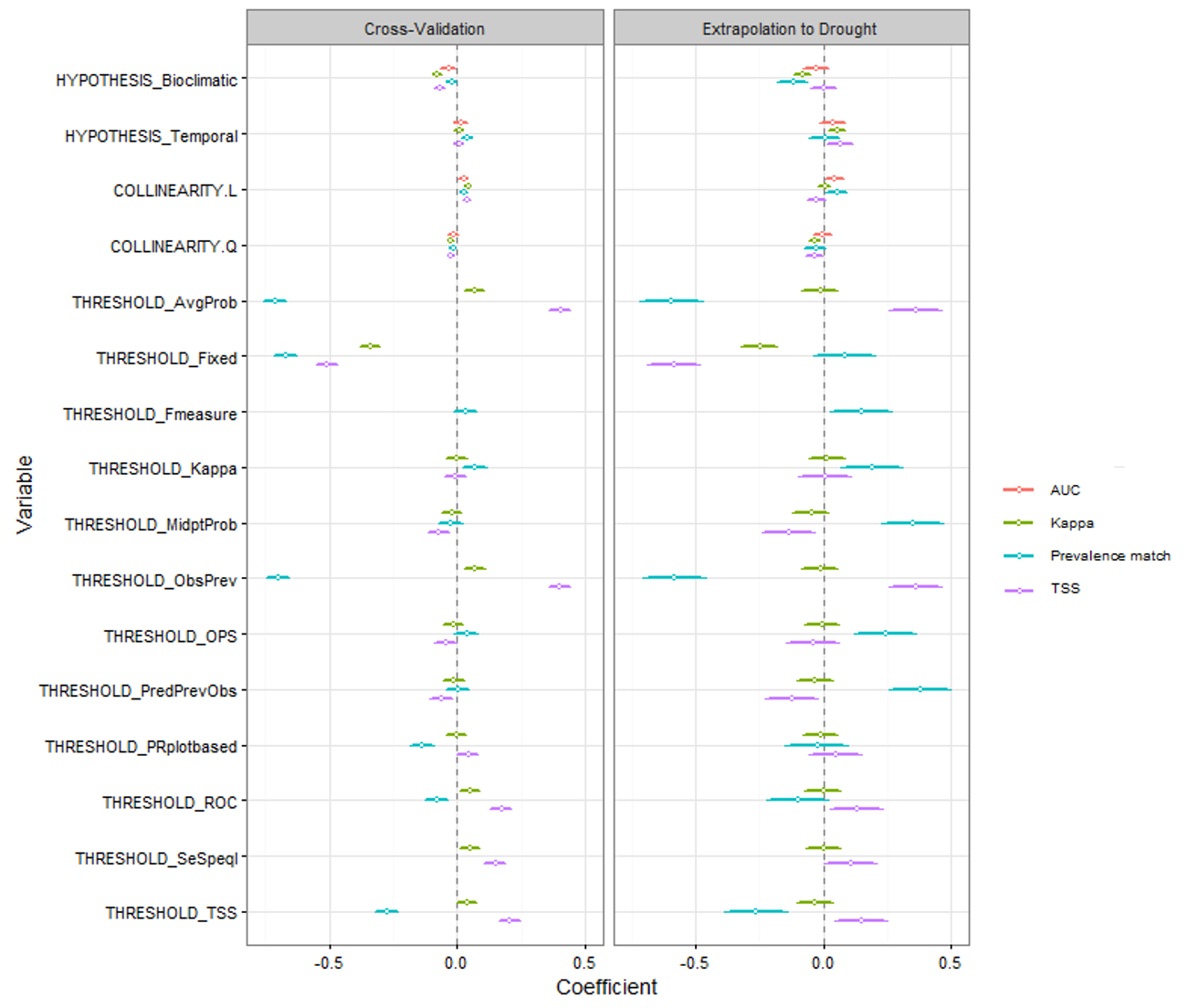

Supplement: Supplementary file 10 [file ECE3-7-8841-s010.tif]
